# Intrahepatic Cholangiocarcinoma with *BRCA* Mutation Achieved Pathological Complete Response after Neoadjuvant Gemcitabine, Cisplatin, and S-1 Therapy: A Case Report

**DOI:** 10.70352/scrj.cr.24-0042

**Published:** 2025-01-31

**Authors:** Yoshifumi Morita, Koki Oda, Akio Matsumoto, Shinya Ida, Ryo Kitajima, Satoru Furuhashi, Makoto Takeda, Hirotoshi Kikuchi, Yoshihiro Hiramatsu, Jun Ito, Takeshi Chida, Hidenao Noritake, Kazuhito Kawata, Yuka Nagakura, Mana Goto, Satoshi Baba, Hiroya Takeuchi

**Affiliations:** 1 Department of Surgery, Hamamatsu University School of Medicine, Hamamatsu, Shizuoka, Japan; 2 Division of Surgical Care, Morimachi, Hamamatsu University School of Medicine, Hamamatsu, Shizuoka, Japan; 3 Department of Perioperative Functioning Care and Support, Hamamatsu University School of Medicine, Hamamatsu, Shizuoka, Japan; 4 Hepatology Division, Department of Internal Medicine II, Hamamatsu University School of Medicine, Hamamatsu, Shizuoka, Japan; 5 Department of Pathology, Hamamatsu University School of Medicine, Hamamatsu, Shizuoka, Japan

**Keywords:** intrahepatic cholangiocarcinoma, *BRCA*, complete response, neoadjuvant, GCS therapy

## Abstract

**INTRODUCTION:**

Intrahepatic cholangiocarcinoma (ICC) is a highly malignant cancer for which surgery is the only curative treatment. The prognosis of ICC is extremely poor, especially in cases of lymph node metastasis (LNM), owing to the high postoperative recurrence rate. Herein, we present a case of advanced ICC with a breast cancer susceptibility gene-2 (*BRCA2*) mutation, treated with preoperative chemotherapy, including cisplatin, followed by surgery, in which we achieved a pathologic complete response.

**CASE PRESENTATION:**

A 52-year-old woman was referred to our hospital and was subsequently diagnosed with bilateral breast cancer. Computed tomography (CT) and magnetic resonance imaging incidentally detected a liver tumor in the hilar region and lymph node enlargement in the hepatoduodenal ligament. A 19 mm tumor was observed in the area surrounded by the right and left branches of the portal vein and an abnormal portal branch of segment 7. Positron emission tomography-CT showed fluorodeoxyglucose uptake in the liver tumor, hepatoduodenal ligament lymph nodes, and bilateral breasts. A tumor biopsy showed a papillary tumor, and ICC was suspected. As ICC with LNM has a poor prognosis, neoadjuvant chemotherapy was planned. Genetic testing using a blood sample revealed a *BRCA2* mutation, indicating the patient would benefit from chemotherapy, particularly cisplatin. The patient received a chemotherapy regimen comprised of gemcitabine, cisplatin, and S-1 (GCS), and after 7 courses, her carbohydrate antigen 19-9 level decreased from 2433 to 15 U/mL. CT showed that the tumor had shrunk and the LNMs were indistinct. The patient was referred to our department for curative surgery, which included a left hepatectomy, caudate lobectomy, hepatoduodenal ligament lymph node dissection, bile duct resection, and choledocojejunostomy. The postoperative course was generally uneventful, and the patient was discharged on postoperative day 18. Pathological examination of the resected specimen revealed an absence of malignant cells. At 24 months postoperative, there was no evidence of recurrence.

**CONCLUSIONS:**

We encountered a patient with advanced ICC with a *BRCA2* mutation, which was successfully treated with preoperative GCS therapy followed by surgical resection, and a pathologic complete response was achieved. GCS therapy, therefore, appears promising as neoadjuvant chemotherapy for the treatment of ICC.

## Abbreviations

ICC
: intrahepatic cholangiocarcinoma
LNM
: lymph node metastasis
BRCA
: breast cancer susceptibility gene
CT
: computed tomography
PET
: positron emission tomography
FDG
: fluorodeoxyglucose
GCS
: gemcitabine, cisplatin, and S-1
BTC
: biliary tract cancer
ERP
: endoscopic retrograde pancreatography

## INTRODUCTION

An increasing high incidence of intrahepatic cholangiocarcinoma (ICC) has been reported in East and Southeast Asia,^1)^ a trend also recently observed in Western countries.^2)^ ICC is highly malignant, and surgery is the only curative treatment. The prognosis of ICC, however, is extremely poor, especially in cases involving lymph node metastasis (LNM) due to a very high recurrence rate after resection.^1)^ Patients with a high risk of recurrence require a multidisciplinary treatment approach, including chemotherapy, radiotherapy, and/or immunotherapy. However, the optimal treatment strategy for advanced ICC has yet to be established. Herein, we present a case of advanced ICC with a breast cancer susceptibility gene-2 (*BRCA2*) mutation. The patient was treated with neoadjuvant chemotherapy, including cisplatin, followed by surgery, which resulted in a pathologic complete response.

## CASE PRESENTATION

**Fig. 1:**
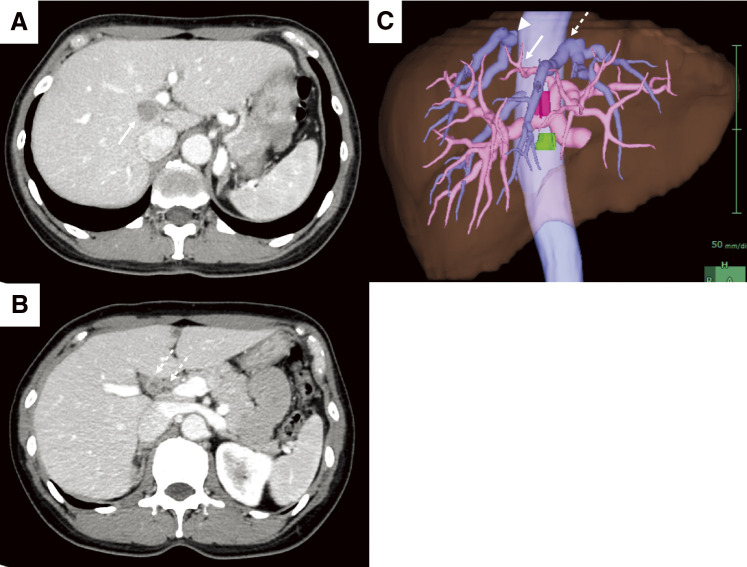
Enhanced CT and 3D reconstruction images before chemotherapy. (**A**) A 19 mm low-density tumor (arrow) is seen at the origin of the right and left branches of the portal vein and the abnormal P7. (**B**) A 12 mm contrast-enhanced lymph node (dotted arrow) is observed in the hepatoduodenal ligament. (**C**) An abnormal portal branch of segment 7 (arrow) branches from the umbilical portion of the portal vein and runs dorsal to the middle hepatic vein (dotted arrow) and the right hepatic vein (arrowhead). The portal vein is shown in pink, and the hepatic vein is shown in light blue. Tumors are shown in red and lymph node metastases are shown in green. CT, computed tomography

**Fig. 2:**
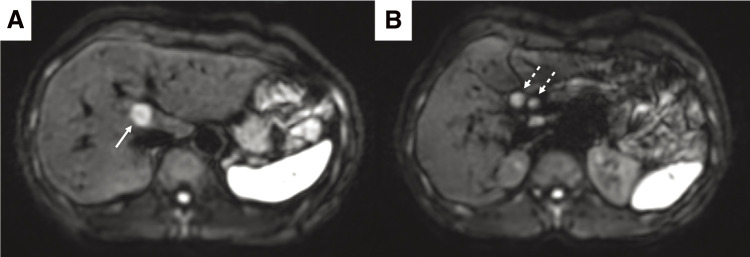
Magnetic resonance imaging findings before chemotherapy. (**A**, **B**) On diffusion-weighted images, both the liver tumor (arrow) and enlarged lymph nodes (dotted arrow) show diffusion restriction.

**Fig. 3:**
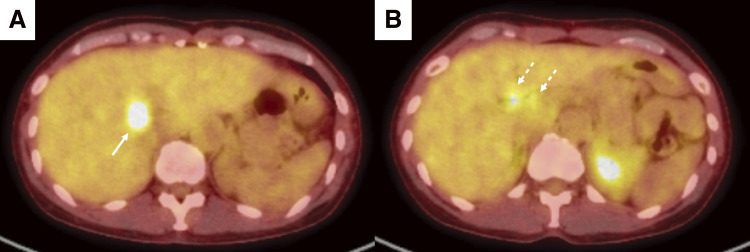
Positron emission tomography-CT findings before chemotherapy. (**A**, **B**) FDG uptake (SUV_max_ = 10.5) is observed in liver tumors (arrow). The lymph nodes (dotted arrow) also show high FDG uptake (SUV_max_ = 5.8). FDG, fluorodeoxyglucose; SUV, standard uptake value

**Fig. 4:**
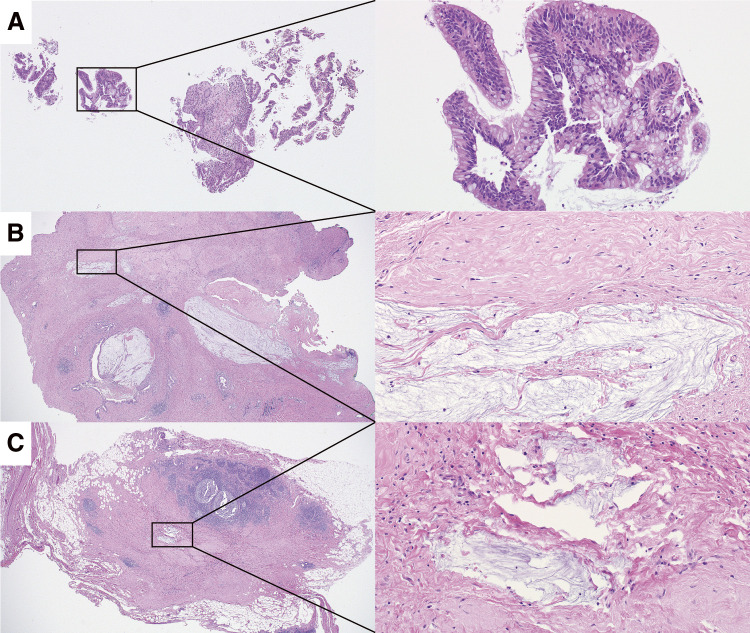
Pathological findings of biopsy and resected specimens. (**A**) Tumor biopsy specimen showing atypical cells with papillary growth that cannot be ruled out as stromal invasion. Left panel: 40× magnification view, right panel: 200× magnification. (**B**) Resected liver specimen showing a scar around the left hepatic bile duct with mucous lakes. No residual carcinoma was observed. Left panel: 20× magnification, right panel: 200× magnification. (**C**) Resected lymph node specimen showing a scar with mucus lakes. No residual carcinoma was observed. Left panel: 20× magnification, right panel: 200× magnification.

**Fig. 5:**
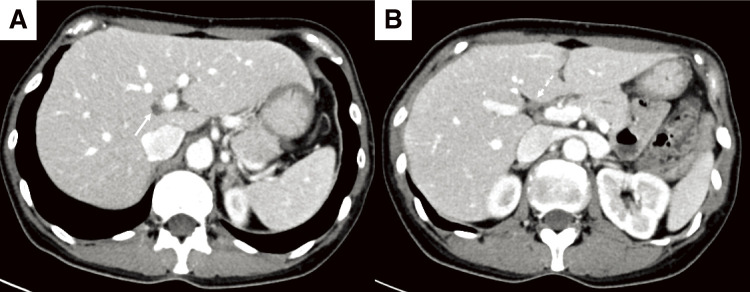
Enhanced CT findings after chemotherapy. (**A**, **B**) Both the liver tumor (arrow) and lymph nodes (dotted arrow) shrank but did not disappear. CT, computed tomography

A 52-year-old woman was referred to our hospital with a right breast calcification on a mammograph. The patient’s family history included gastric and esophageal cancer in his mother. After a thorough examination, including a biopsy, the patient was diagnosed with bilateral breast cancer. Computed tomography (CT) and magnetic resonance imaging incidentally revealed a liver tumor in the hilar region (**Figs. 1A** and **2A**), as well as enlarged lymph nodes in the hepatoduodenal ligament (**Figs. 1B** and **2B**). Additionally, there was an abnormal portal branch of segment 7 (P7) that originated from the umbilical portion of the portal vein. A 19 mm tumor was observed to be surrounded by the right and left branches of the portal vein and the abnormal P7 (**Fig. 1C**). Positron emission tomography (PET)-CT showed fluorodeoxyglucose uptake in the liver tumors, hepatoduodenal ligament lymph nodes (**Fig. 3A**), and bilateral breasts, although no uptake was observed in the axillary lymph nodes. The patient’s liver function was normal and there was no evidence of hepatitis virus infection or history of alcohol consumption. A tumor biopsy showed a papillary tumor, and ICC was suspected (**Fig. 4A**). The clinical diagnosis was T1aN1M0 stage IIIa, according to the Union for International Cancer Control TNM Classification of Malignant Tumours, 8th edition.^3)^ Because ICC with LNMs has a poor prognosis, we planned to administer neoadjuvant chemotherapy. Since the patient had bilateral breast cancer, we searched for *BRCA* germline mutations. Genetic testing using a blood sample revealed a *BRCA2* mutation, indicating the patient would benefit from chemotherapy, particularly cisplatin. A chemotherapy regimen comprised of gemcitabine, cisplatin, and S-1 (GCS) was selected for neoadjuvant chemotherapy. The patient received systemic chemotherapy with gemcitabine (1000 mg/m^2^) and cisplatin (25 mg/m^2^) infused on days 1 and 8, while S-1 was administered orally (120 mg/day) from days 1 to 7. Grade 3 diarrhea was observed after four courses of GCS; therefore, the dose of S-1 was reduced to 80 mg/day and chemotherapy was continued. After 7 courses of GCS therapy, the serum carcinoembryonic antigen level decreased from 6.0 to 2.5 ng/mL and the carbohydrate antigen 19-9 level decreased from 2433 to 15 U/mL. A post-chemotherapy CT scan showed that the tumor had shrunk and the LNMs had become indistinct, although they did not disappear (**Fig. 5**). The patient was referred to our department for curative surgery. Because left hepatectomy can create a large ischemic area in the right posterior segment of the remnant liver, the left portal vein and abnormal P7 were preoperatively embolized. The expected future remnant liver volume increased from 45% to 50%. Subsequently, we performed curative surgery which included a left hepatectomy, caudate lobectomy, lymph node dissection around the hepatoduodenal ligament, bile duct resection, and choledocojejunostomy. The abnormal G7 appeared behind the middle hepatic vein on the liver resection line. The abnormal G7 was ligated and dissected, but no obvious ischemic area was observed. The root of the left hepatic duct was confirmed at the hepatic hilum, and the right hepatic duct was preserved. The operative time was 553 min, and the intraoperative blood loss was 513 mL. The postoperative course was generally uneventful, and the patient was discharged on postoperative day 18. Pathological examination of the resected liver specimen revealed an absence of malignant cells. Pathological examination of the resected lymph nodes in the hepatoduodenal ligament and posterior pancreatic head revealed scar-like fibrotic nests, again with an absence of malignant cells (**Figs. 4B** and **4C**). These pathological results suggested that the tumor disappeared because of the effects of the neoadjuvant GCS therapy. The patient underwent bilateral nipple-sparing mastectomies 17 months after surgery, and after 24 months, the patient remained well with no evidence of tumor recurrence.

## DISCUSSION

In the case presented herein, neoadjuvant GCS therapy was administered in the treatment of ICC with multiple LNMs, and postoperative pathology confirmed a complete response. The patient had a *BRCA2* mutation, which may have contributed to the positive response to the cisplatin-containing chemotherapy regimen.

The incidence of LNM in ICC is reported to be approximately 30%–53% for mass-forming ICC and more than 60% for periductal-infiltrating or mass-forming plus periductal-infiltrating ICC.^4)^ Regarding clinical implications, routine lymphadenectomy has been considered beneficial for accurate staging and for guiding postoperative treatment strategies. As such, an international consensus conference has recommended that lymph node dissection should be considered in the surgical treatment of ICC.^5)^ The efficacy of prophylactic lymph node dissection on long-term survival, however, is unclear in cases where no LNMs were detected on preoperative imaging.^6)^ A collaborative Korean-Japanese study revealed that the number of LNMs was associated with poor prognosis and that the surgical retrieval of ≥ 4 lymph nodes was associated with improved survival outcomes in patients with ICC and LNM.^7)^ The concept of lymph node dissection based on tumor location and risk of LNM has been recently proposed,^8)^ while at present, the initial treatment for patients with numerous LNMs is preoperative systemic chemotherapy followed by restaging to ensure the absence of disease progression.^1)^

The effectiveness of chemotherapy in all patients with ICC remains controversial. A retrospective analysis of a national clinical database in the United States did not confirm the benefits of neoadjuvant chemotherapy for the treatment of resectable ICC.^9)^ Neoadjuvant chemotherapy has been associated with a lower rate of LNMs but has not conferred a survival benefit compared with upfront surgery. This retrospective study, however, did not confirm the chemotherapy regimen, nor did it report on the completion of chemotherapy.

GCS demonstrated superior efficacy compared to GC in terms of overall survival (OS) (median OS: 13.5 months with GCS vs. 12.6 months with GC; hazard ratio [HR] = 0.79; 90% confidence interval [CI] = 0.628–0.996; *P* = 0.046). In particular, the objective response rates were 41.5% and 15.0% in the GCS and GC arms, respectively (*P* <0.001).^10)^ This response rate is very high compared with that of conventional chemotherapy and is promising for the effectiveness of neoadjuvant chemotherapy. Few cases have been reported of primary unresectable ICC with distant LNMs being successfully treated by conversion surgery following neoadjuvant GCS.^11,12)^ A Phase II trial (NCT01821248, KHBO1201) was recently initiated to evaluate the efficacy and safety of neoadjuvant GCS in patients with biliary tract cancer (BTC) and LNM. In the present case, immunotherapy for BTC was not covered by the Japanese health insurance system. Recently, GC plus durvalumab and GC plus pembrolizumab have been covered by insurance for unresectable or recurrent BTC. With recent advancements in molecular targeted therapy and immunotherapy, significant progress in neoadjuvant cancer treatment is expected in the future.^13,14)^

Approximately 40% of BTC cases have potentially targetable genetic alterations. *BRCA2* mutations have been detected in up to 5% of all BTC cases. *BRCA1* mutations occur in 1% and *BRCA2* mutations occur in 4% of patients with ICC.^15)^ *BRCA* mutations have been used as biomarkers to predict the sensitivity to deoxyribonucleic acid-damaging therapies, platinum-based alkylating agents, in particular.^16)^ Therefore, GCS therapy appears to be a particularly promising preoperative treatment for patients with ICC and *BRCA* mutations. Because the patient had bilateral breast cancer simultaneously, we could search for *BRCA* germline mutations before starting preoperative treatment. In the present case, even if the patient had wild-type *BRCA*, the GCS regimen with a high response rate would have been chosen. Although the Japanese insurance system allows multigene panel testing only for unresectable or recurrent patients, the indication should be expanded for highly selected BTC patients.

Postoperative adjuvant chemotherapy is also important for patients with ICC who have a high risk of recurrence. Based on the results of a randomized Phase III study of S-1 vs. observation in patients with curatively resected BTCs (JCOG1202: ASCOT), adjuvant S-1 therapy has become the standard treatment in Japan, as well as several other Asian countries.^17)^ In Western countries, capecitabine has been recommended for 6 months as adjuvant chemotherapy following surgery for patients with resected BTC.^18)^ In the case presented herein, a postoperative examination confirmed a pathologic complete response. The patient was subsequently scheduled for breast cancer resection; therefore, no adjuvant chemotherapy was administered.

## CONCLUSION

In conclusion, we encountered a patient with advanced ICC and a *BRCA2* mutation that was successfully treated with neoadjuvant GCS therapy followed by surgical resection, after which a pathologic complete response was achieved. GCS therapy has a high response rate and appears promising as neoadjuvant chemotherapy, particularly in patients with *BRCA* mutations.

## ACKNOWLEDGMENTS

None.

## DECLARATIONS

### Funding

No funding was received for this report.

### Authors’ contributions

YM wrote the first draft of the manuscript.

All authors commented on the subsequent versions of the manuscript.

All authors have read and approved the final version of the manuscript.

### Availability of data and materials

Not applicable.

### Ethics approval and consent to participate

The case report was approved by the ethical committee of Hamamatsu University School of Medicine (approval number: 17-124).

### Consent for publication

Informed consent was obtained from the patient for the publication of this case report.

### Competing interests

The authors have no competing interests to declare that are relevant to the content of this article.
